# Editorial: New insights: developments in laboratory testing for the diagnosis and monitoring of endocrine related disorders and metabolic disease

**DOI:** 10.3389/fendo.2024.1524950

**Published:** 2024-11-29

**Authors:** Verena Gounden, Pranoot Tanpaiboon

**Affiliations:** ^1^ Department of Chemical Pathology, University of KwaZulu Natal, National Health Laboratory Service (NHLS), Johannesburg, South Africa; ^2^ Department of Biochemical Genetics, Quest Diagnostics (United States), Madison, NJ, United States

**Keywords:** laboratory investigation, endocrine disorders, mass- spectrometry, chip technology, non-alcocholic fatty liver disease

Laboratory investigations remain one of the primary tools utilized in the diagnosis and monitoring of endocrine pathologies and metabolic disease. The development of the first immunoassay by Nobel winners Berson and Yalow in 1959 was revolutionary in providing diagnostic testing for the spectrum of endocrine related disorders. Whilst immunoassays remain a cornerstone of laboratory analytics, there have been several significant improvements in this technology and addition of various other techniques such as tandem- mass spectrometry for routine investigation of endocrine and metabolic disease (1). In the midst of the fourth revolution advances in computing and artificial intelligence have already made their way into the medical laboratory and will play a growing role in patient testing and the way laboratories operate.

The aim of this Research Topic was to report and highlight recent developments in laboratory testing specific to the diagnosis, monitoring and evaluation of endocrine related disorders and metabolic disease. The published reports of this Research Topic ranged across the wide landscape of laboratory methodologies. This Research Topic examines the use of predictive models and existing routine lab investigations utilised in new ways to detect non-alcoholic fatty liver disease (NAFLD) and liver fibrosis; the development of the immunometric measurement of new biomarkers such as vasoinhibin; as well as use of chip technology and secondary electrospray ionisation high- resolution mass spectrometry (SESI-HRMS) for metabolomic evaluation.

NAFLD or most recently referred to as metabolic dysfunction- associated steatotic liver disease refers to a disease continuum from steatosis with or without inflammation to steatohepatitis to fibrosis and cirrhosis. Estimates indicate that approximately 25% of the global population has this condition and it is rapidly on the increase in children and adolescents (2). There are three studies reporting the association between NAFLD and liver fibrosis with different lipoprotein markers/ratios. These studies used the same data source, the National Health and Nutrition Examination Survey (NHANES), however, the data was extracted from different periods. In this Research Topic, Guo et al. report the development and validation of a non-invasive predictive model for the assessment of significant fibrosis in patients with NAFLD. Their new model, that was established using data extracted from the NHANES database, utilises nine common and easily accessible indicators such as BMI and routine laboratory tests such as alanine aminotransferase (ALT) and high-density lipoprotein cholesterol (HDLC). The model performed well when compared to current in use scoring systems achieving the highest AUC of 0.802 with a specificity of 0.823. Two groups utilised different lipid markers to assess the relationship between NAFLD and liver fibrosis. Wang et al. and Xuan et al. reporting their findings regarding the use of different lipoprotein markers in this Research Topic. Wang et al. investigated the link between the triglyceride to HDLC ratio in NAFLD. Whilst Xuan et al. examined the association of the non-HDLC to HDLC ratio to NAFLD and liver fibrosis. Both studies highlight the utility of these markers and predictive and severity markers in this disease process. Further prospective studies are needed to demonstrate causality and not only association as demonstrated in the cross- sectional analyses by Wang et al. and Xuan et al.

This Research Topic also explores developments in laboratory testing in regards to diabetes mellitus. Diabetes remains a significant cause of mortality and morbidity worldwide, with the growing incidence in developing nations being of significant concern (3). There are 2 exploratory studies related to diabetes mellitus in this Research Topic Awchi et al. present a very interesting study investigating the feasibility of using breath analysis to monitor changes in metabolites to assess the course of acute diabetic acidosis. Whilst a small study this paper provides important proof of concept for this type of non-invasive evaluation using SESI-HRMS. Diabetes is the leading cause of chronic kidney disease. In their article, Li et al. demonstrate the simultaneous measurement of multiple urine biomarkers for the early prediction of diabetic kidney disease. They utilised chip detection technology to evaluate these markers. Use of such technology is likely to have increased scope in the near-patient testing environment or in health care facilities where laboratory access is limited due to geographical and logistical obstacles.

After diabetes mellitus, thyroid- related disorders represent the second most common group of endocrine disorders (4). In autoimmune thyroid diseases, such as Graves disease or Hashimotos thyroiditis, currently available assays whilst able to quantitate the presence of TSH- receptor antibodies have been unable to reliably distinguish between activating and blocking antibodies. In their paper, George et al. describe their development of a novel bioassay for the detection of thyroid blocking immunoglobulins.

In this Research Topic, the role of the laboratory investigations in reproductive endocrinology has also been explored. Huang et al. systematically evaluated the utility of serum matrix metalloproteinase -9 (MMP-9) as diagnostic marker for endometriosis. Their findings show promise for the use of a single marker that would be able to reliably detect the presence of a condition such as endometriosis, which is extremely heterogeneous in presentation and severity.

Measurement of the antiangiogenic protein vasoinhibin is of interest in vasoproliferative retinopathies, preeclampsia, and peripartum cardiomyopathy. In this Research Topic, Zamora et al. describe the development of a novel ELISA method for the detection of this hormone. They highlight the versatility of immunoassay methodologies for not just routine hormone quantitation but also for the detection of novel biomarkers.

The clinical laboratory plays important role in the diagnosis, monitoring of disease and treatment, identification of pre-symptomatic disease, as well as risk prediction. The rapid evolution of knowledge and technology have helped us to identify new biomarkers and new diagnostic methods. However, currently available biomarkers with new algorithms can be repurposed to aid both the laboratorian and the clinician to improve patient management.

Finally, we would like to thank all our contributors whose manuscripts have added significant value to this Research Topic.

## References

[1] Wu AHB. A selected history and future of immunoassay development and applications in clinical chemistry. Clinica Chimica Acta. (2006) 369:119–24. doi: 10.1016/j.cca.2006.02.045 16701599

[2] Powell EE Wong VW Rinella M. Non-alcoholic fatty liver disease. Lancet. (2021) 397:2212–24. doi: 10.1016/S0140-6736(20)32511-3 33894145

[3] International Diabetes Federation. Diabetes fact and figures. Available online at: https://idf.org/about-diabetes/diabetes-facts-figures/ (Accessed 7 November 2024).

[4] Taylor PN Albrecht D Scholz A Gutierrez-Buey G Lazarus JH Dayan CM. Global epidemiology of hyperthyroidism and hypothyroidism. Nat Rev Endocrinol. (2018) 14:301–16. doi: 10.1038/nrendo.2018.18 29569622

